# Alzheimer’s Disease—A Panorama Glimpse 

**DOI:** 10.3390/ijms150712631

**Published:** 2014-07-16

**Authors:** Li Na Zhao, Lanyuan Lu, Lock Yue Chew, Yuguang Mu

**Affiliations:** 1 School of Physical and Mathematical Sciences, Nanyang Technological University, 21 Nanyang Link, 637731, Singapore; E-Mail: zhao0139@e.ntu.edu.sg; 2 School of Computer Engineering, Nanyang Technological University, 50 Nanyang Avenue, 639798, Singapore; 3 School of Biological Sciences, Nanyang Technological University, 60 Nanyang Drive, 637551, Singapore; E-Mail: lylu@ntu.edu.sg

**Keywords:** Alzheimer’s disease, amyloid *β* peptide, amyloid *β* oligomer, tau protein, A*β* variants, A*β* polymorphism, pyroglutamate-modified amyloid beta peptides

## Abstract

The single-mutation of genes associated with Alzheimer’s disease (AD) increases the production of A*β* peptides. An elevated concentration of A*β* peptides is prone to aggregation into oligomers and further deposition as plaque. A*β* plaques and neurofibrillary tangles are two hallmarks of AD. In this review, we provide a broad overview of the diverses sources that could lead to AD, which include genetic origins, A*β* peptides and tau protein. We shall discuss on tau protein and tau accumulation, which result in neurofibrillary tangles. We detail the mechanisms of A*β* aggregation, fibril formation and its polymorphism. We then show the possible links between A*β* and tau pathology. Furthermore, we summarize the structural data of A*β* and its precursor protein obtained via Nuclear Magnetic Resonance (NMR) or X-ray crystallography. At the end, we go through the *C*-terminal and *N*-terminal truncated A*β* variants. We wish to draw reader’s attention to two predominant and toxic A*β* species, namely A*β*_4−42_/ and pyroglutamate amyloid-beta peptides, which have been neglected for more than a decade and may be crucial in A*β* pathogenesis due to their dominant presence in the AD brain.

## 1. Introduction

Alzheimer’s disease was first recognized by Alois Alzheimer as presenile dementia in 1906. It is mainly diagnosed in people whose ages are over 65 with the prevalence of Alzheimer’s Disease (AD) being shown to grow exponentially with age. It is prevalent among 10% of elderly people, which makes AD an emerging social health issue with the rise of an aging population in the coming decades. Specially, at the age of 85, 50% of the people face the risk of developing AD [1,2]. However, AD is not exactly an aging-related disease [3]. In fact, it has been classified into two types. One is the gene-related heritable AD, known as the early onset familial Alzheimer’s disease (fAD). The clinical symptoms can appear in a very young age and it accounts for 25% of all AD cases [4]. Another type is the sporadic Alzheimer’s disease (sAD), which constitutes the vast majority of AD cases and is also apparently influenced by genetic contributions besides non-genetic environmental factors [5,6,7]. Several genes have been identified to increase the chance of developing fAD and sAD. However, the pathological role of the only identified *є*4 *lipoprotein E* (APOE) gene in sAD is still unclear. In addition, the mutation of APOE *є*4 is not necessary to increase the risk of developing sAD [8,9]. Thus, the late-onset AD degenerative process has been speculated to be polygenic with the involvement of multiple risk factors [6]. 

It is well known that the early symptoms of AD include loss of short-term memory, difficulties in executing daily life activities, and withdrawal from social life. The behavioral symptoms include progressive decline in memory, spatial reasoning, attention, and languages. AD is mainly characterized by two pathological hallmarks: the intracellular neurofibrillary tangle (NFT) formed by hyperphosphorylated tau proteins, and the extracellular amyloid plaque consisting of amyloid *β* peptides. Significant selective neuronal degeneration and loss, with neurotransmitter deficits and inflammations are also evident [10]. In the following section, we first delve into the root of AD by giving a short review on genetic risk factors. After which, we shall discuss on two focal areas of current AD research. One involves the intracellular accumulation of tau protein while the other is on extracellular amyloid aggregation. The former is covered in Section 3 and the latter is addressed in Section 4, Section 5 and Section 6. In Section 7, we connect these two aspects of AD research to provide a panoramic view of the generative mechanism of AD. We briefly discuss on the progression pathway of AD and the possible therapeutic approaches in Section 8. Finally, we conclude our review in Section 9. 

## 2. Genetic Revelation of AD

With the higher level of gene expression in the brain, the cumulative DNA damage may have a cascading effect on the transcriptional effectivity and fidelity, and the alteration of DNA conformation in the hippocampus region has been observed in the brain of AD patients [11,12]. For the two types of Alzheimer’s—early onset (a.k.s. familial AD) and later onset (a.k.s. sporadic AD), both have a genetic connection. Familial AD involves a number of single-gene mutations on chromosomes 1, 14 and 21, which corresponds to the abnormal presenilin 2, presenilin 1 and amyloid precursor protein production respectively [13,14,15,16,17], and each of these mutations is believed to play a very important role in the cleavage of APP and thus affect A*β* production. The late-onset AD accounts for the major cases of AD, notwithstanding a lack of full understanding, the genetic risk factors, such as the definitively identified *apolipoprotein E* (*APOE*) gene on chromosome 19 [18] and the *methylenetetrahydrofolate dehydrogenase 1-like* (*MTHFD1L*) gene on chromosome 6 [19] as well as some other loci [20,21,22,23], are likely to affect the predisposition of AD. Additionally, the genome-wide study of AD cases also revealed new novel variants, which may modify the age of the AD onset or show gender-linked susceptibility [24,25]. 

## 3. Tau Protein and Tau Accumulation

Tau protein arises from the alternative splicing of the *microtubule-associated protein tau* (*MAPT*) gene, and is abundant within the central and peripheral nervous systems. It is one of the intrinsically unstructured proteins (IUPs), which exhibit as random coils under physiological conditions, and are capable of folding into well-defined stable structures, e.g., the neurofibrillary tangles in AD. Normally, it is the phosphorylation-modified tau protein that stabilizes the axonal microtubules in the central nervous system (CNS). Moreover, tau protein with actin cytoskeleton and plasma membrane serve as enzyme anchors, and they are also believed to help in the neurite outgrowth and the transport of axoplasm. However, under certain circumstances, tau protein may undergo abnormal phoshorylation, hyperphosphorylation and some other modifications -nitration, ubiquitination, truncation, shift, prolyl isomerization, which may reduce the binding affinity of tau towards microtube, and thus lead to either intraneuronal accumulation of tau protein or its binding to other macromolecules [26,27,28,29]. 

The mislocalization and accumulation of tau proteins in dendrites and dendritic spines brings about a disruption of neuronal cell communication, which precedes neurodegeneration and causes a loss of memory [30]. Tau self-assemblies of tau proteins forming straight filaments (SFs) and/or paired helical filaments (PHF) may further aggregate into NFT, which is significantly correlated with the severity of AD. Electron microscopy of PHFs showed the appearance of two strands twisting around each other with a cross-over repeat of 75–80 nm and a width of 10–22 nm [31,32]. It has also been further revealed that *β*-sheet is the most dominant structure in the PHFs. 

## 4. Amyloid Plaque and A*β* Peptide

### 4.1. Amyloid Precursor Protein

The amyloid *β* plaques results from the aggregation of the amyloid *β* (A*β*) peptides, which is cleaved by the *β*-and γ-secreastase from the amyloid precursor protein (APP). The precise biological function of APP is as yet not well defined even though lots of studies have revealed its biological and physiological importance in the neurite outgrowth modulation [33], copper homeostasis regulation [34], synaptic transmission and formation, and synaptic function and activity [35,36]. On the other hand, it has been shown that the absence of APP in a mouse model did not cause a significant impairment of cognitive abilities, but instead led to a decrease in locomotion activity [37]. 

### 4.2. A*β* Aggregation Pathway

Neuronal impairment is observed in patients even before A*β* plaque formation during the early onset AD. It is generally believed that A*β* oligomer is the main culprit of neurotoxicity [38,39,40,41]. Due to these oligomers being easily attached to the membrane or other macromolecules and hard to be isolated from these structures, conventional experimental studies on them are very difficult [42]. A progress has been made recently which shows that A*β* dimers can be measured and strongly associated with dementia [43]. Besides the experimental methods, molecular dynamic simulation is a complementary approach for atomic-level studies of the unstructured monomer aggregation process, structural evolution and toxicity. 

### 4.3. Structural Evolution during Aggregation

In recent decades, Nuclear Magnetic Resonance (NMR) and X-ray have been carried out to determine the structures of A*β* in water and membrane mimic environment (see Table 1). One general observation is that A*β* peptides exhibit great polymorphism. 

**Table 1:** Structural data of amyloid beta peptide obtained from experiments.

PDB ID	Experimental Technique	Resolution	Release Date	Residue	DOI
1AMB [44]	SOLUTION NMR	-	1994-12-20	1–28	-
1AMC [44]	SOLUTION NMR	-	1995-01-26	1–28	-
1AML [45]	SOLUTION NMR	-	1996-01-29	1–40	-
1BA4 [46]	SOLUTION NMR	-	1998-06-17	1–40	-
1BA6 [47]	SOLUTION NMR	-	1998-06-17	1–40	10.1021/bi972979f
1BJB [48]	SOLUTION NMR	-	1998-11-04	1–28	10.1006/jsbi.2000.4267
1BJC [48]	SOLUTION NMR	-	1998-11-18	1–28	10.1006/jsbi.2000.4267
1HZ3 [49]	SOLUTION NMR	-	2001-01-31	1–26	10.1002/pro.5560060902
1IYT [50]	SOLUTION NMR	-	2003-02-11	1–42	10.1006/jsbi.2000.4288
1NMJ [51]	SOLUTION NMR	-	2003-01-28	1–28	10.1038/7562
1QWP [52]	SOLUTION NMR	-	1997-07-07	25–35	10.1021/bi961598j
1QXC [52]	SOLUTION NMR	-	2004-09-14	25–35	-
1QYT [52]	SOLUTION NMR	-	2004-12-14	25–35	10.1021/jm040773o
1ZE7 [53]	SOLUTION NMR	-	2005-05-03	1–16	10.1074/jbc.M504454200
2BP4 [53]	SOLUTION NMR	-	2005-04-21	1–16	10.1074/jbc.M504454200
2LI9 [54]	SOLUTION NMR	-	2011-07-20	1–16	10.1016/j.bbrc.2011.06.133
2LI9 [54]	SOLUTION NMR	-	2012-01-18	1–16	-
2M9S [55]	SOLUTION NMR	-	-	1–40	10.1016/j.bbagen.2013.06.031
2Y2A [56]	X-RAY DIFFRACTION	-	2011-10-26	16–21	10.1073/pnas.1112600108
2Y3J [56]	X-RAY DIFFRACTION	1.91	-	30–35	10.1073/pnas.1112600108
2Y3L [56]	X-RAY DIFFRACTION	2.1	2011-11-02	35–42	10.1073/pnas.1112600108
2Y3K [56]	X-RAY DIFFRACTION	2.1	2011-11-02	35–42	10.1073/pnas.1112600108
3Q2X [56]	X-RAY DIFFRACTION	2.1	2011-11-02	27–32	10.1073/pnas.1112600108
3PZZ [56]	X-RAY DIFFRACTION	2.1	2011-11-02	29–34	10.1073/pnas.1112600108
3OVJ [57]	X-RAY DIFFRACTION	1.8	2011-07-06	16–21	10.1016/j.molcel.2004.06.037
3OW9 [56]	X-RAY DIFFRACTION	1.8	2011-08-31	16–21	10.1371/journal.pbio.1001080
	SOLUTION NMR	-	2004-09-14	25–35	10.1021/jm040773o
	SOLUTION NMR	-	2012-01-18	1–16	10.1016/j.bpj.2011.11.4006
2OTK [58]	3D NMR	-	2013-09-11	1–40	10.1016/j.bbagen.2013.06.031

In many situations, A*β* is found to easily attach to other protein, or self-assemble into large oligomers or fibrils. These complex involving A*β* peptides, such as APP, A*β* binding copper ions, A*β* segments with enzymes as observed in experiment, are cataloged in Table 2. 

**Table 2:** Experimental data of amyloid beta oligomers, fibrills and binding proteins.

PDB ID	Experimental Technique	Resolution	Release Date	Residues Count	DOI
1AAP [59]	X-RAY DIFFRACTION	1.5	1991-10-15	116	-
1BRC [60]	X-RAY DIFFRACTION	2.5	1994-05-31	279	10.1006/jmbi.1993.1211
1TAW [61]	X-RAY DIFFRACTION	1.8	1997-06-24	281	10.1002/pro.5560060902
1CA0 [61]	X-RAY DIFFRACTION	2.1	1997-07-23	590	10.1002/pro.5560060902
1X11 [62]	X-RAY DIFFRACTION	2.5	1998-01-14	370	10.1093/emboj/16.20.6141
1MWP [63]	X-RAY DIFFRACTION	1.8	2000-03-15	96	10.1038/7562
1OQN [64]	X-RAY DIFFRACTION	2.3	2003-08-05	336	10.1074/jbc.M304384200
1OWT [65]	SOLUTION NMR	-	2003-05-13	66	10.1074/jbc.M300629200
1TKN [66]	SOLUTION NMR	-	2004-08-03	110	10.1021/bi049041o
1ZJD [67]	X-RAY DIFFRACTION	2.6	2005-08-09	294	10.1074/jbc.M504990200
2BEG [68]	SOLUTION NMR	-	2005-11-22	210	10.1073/pnas.0506723102
2G47 [69]	X-RAY DIFFRACTION	2.1	2006-10-24	2060	10.1038/nature05143
2FJZ [70]	X-RAY DIFFRACTION	1.61	2007-01-16	59	10.1016/j.jmb.2006.12.041
2FK1 [70]	X-RAY DIFFRACTION	1.6	2007-01-16	59	10.1016/j.jmb.2006.12.041
2FK2 [70]	X-RAY DIFFRACTION	1.65	2007-01-16	59	10.1016/j.jmb.2006.12.041
2FK3 [70]	X-RAY DIFFRACTION	2.4	2007-01-16	472	10.1016/j.jmb.2006.12.041
2FKL [70]	X-RAY DIFFRACTION	2.5	2007-01-16	132	10.1016/j.jmb.2006.12.041
2FMA [71]	X-RAY DIFFRACTION	0.85	2007-01-16	59	10.1107/S1744309107041139
2IPU [72]	X-RAY DIFFRACTION	1.65	2007-10-09	906	10.1073/pnas.0705888104
2R0W [72]	X-RAY DIFFRACTION	2.5	2007-10-16	450	10.1073/pnas.0705888104
2OTK [58]	SOLUTION NMR	-	2008-02-12	182	10.1073/pnas.0711731105
2ROZ [73]	SOLUTION NMR	-	2008-07-22	168	10.1074/jbc.M803892200
3BAE [74]	X-RAY DIFFRACTION	1.59	2008-04-15	474	10.1016/j.jmb.2007.12.036
3BKJ [74]	X-RAY DIFFRACTION	1.59	2008-04-15	492	10.1016/j.jmb.2007.12.036
3DXC [75]	X-RAY DIFFRACTION	2.1	2008-09-16	350	10.1038/embor.2008.188
3DXD [75]	X-RAY DIFFRACTION	2.2	2008-09-16	350	10.1038/embor.2008.188
3DXE [75]	X-RAY DIFFRACTION	2.0	2008-09-16	350	10.1038/embor.2008.188
3GCI [76]	X-RAY DIFFRACTION	2.04	2009-03-10	126	-
3IFL [77]	X-RAY DIFFRACTION	1.5	2009-11-17	448	10.1074/jbc.M109.045187
3IFN [77]	X-RAY DIFFRACTION	1.5	2009-11-17	481	10.1074/jbc.M109.045187
3IFO [77]	X-RAY DIFFRACTION	2.15	2009-11-17	904	10.1074/jbc.M109.045187
3IFP [77]	X-RAY DIFFRACTION	2.95	2009-11-17	1808	10.1074/jbc.M109.045187
3JQ5 [76]	X-RAY DIFFRACTION	2.03	2009-09-29	127	-
3JQL [76]	X-RAY DIFFRACTION	1.2	2009-09-29	125	-
2WK3 [78]	X-RAY DIFFRACTION	2.59	2009-11-03	2122	10.1016/j.jmb.2009.10.072
3KTM [79]	X-RAY DIFFRACTION	2.7	2010-02-23	1528	10.1073/pnas.0911326107
3L81 [80]	X-RAY DIFFRACTION	1.6	2010-06-02	308	10.1016/j.devcel.2010.01.015
3JTI [81]	X-RAY DIFFRACTION	1.8	2010-07-21	127	-
3L33 [82]	X-RAY DIFFRACTION	2.48	2010-09-22	1104	10.1074/jbc.M110.171348
3MOQ [83]	X-RAY DIFFRACTION	2.05	2011-02-16	504	10.1523/JNEUROSCI.4259-10.2011
3MXC [84]	X-RAY DIFFRACTION	2.0	2011-05-11	110	10.1016/j.jmb.2011.09.046
3NYJ [85]	X-RAY DIFFRACTION	3.2	2011-06-01	207	10.1021/bi101846x
3NYL [86]	X-RAY DIFFRACTION	2.8	2011-07-13	210	10.1016/j.molcel.2004.06.037
2Y3J [87]	X-RAY DIFFRACTION	1.99	2011-11-02	48	10.1073/pnas.1112600108
2Y3K [87]	X-RAY DIFFRACTION	1.9	2011-11-02	64	10.1073/pnas.1112600108
3AYU [88]	X-RAY DIFFRACTION	2.0	2011-08-03	177	10.1074/jbc.M111.264176
2LMN [89]	SOLID-STATE NMR	-	2011-12-28	480	10.1021/bi051952q
2LMO [89]	SOLID-STATE NMR	-	2011-12-28	480	10.1021/bi051952q
2LMP [90]	SOLID-STATE NMR	-	2011-12-28	720	10.1073/pnas.0806270105
2LMQ [90]	SOLID-STATE NMR	-	2011-12-28	720	10.1073/pnas.0806270105
2LNQ [91]	SOLID-STATE NMR	-	2012-02-08	320	10.1073/pnas.1111305109
2LOH [92]	SOLUTION NMR	-	2012-05-23	86	10.1016/j.febslet.2012.04.062
2LP1 [93]	SOLUTION NMR	-	2012-06-06	122	10.1126/science.1219988
2LLM [94]	SOLUTION NMR	-	2012-06-20	43	PMCID: PMC3347594
3U0T [95]	X-RAY DIFFRACTION	2.5	2012-01-11	894	10.1016/j.jmb.2011.11.047
3UMH [96]	X-RAY DIFFRACTION	2.0	2012-01-25	211	10.1016/j.jmb.2011.12.057
3UMI [96]	X-RAY DIFFRACTION	2.4	2012-01-25	211	10.1016/j.jmb.2011.12.057
3UMK [96]	X-RAY DIFFRACTION	2.6	2012-01-25	211	10.1016/j.jmb.2011.12.057
3SV1 [97]	X-RAY DIFFRACTION	3.3	2012-07-11	612	10.1093/jmcb/mjs033
4HIX [98]	X-RAY DIFFRACTION	2.2	2013-03-13	475	10.1038/srep01302
2M4J [99]	SOLUTION NMR	-	2013-09-25	360	10.1016/j.cell.2013.08.035
2LZ3 [100]	SOLUTION NMR	-	2013-10-02	56	-
2LZ4 [100]	SOLUTION NMR	-	2013-10-02	56	-

The central amyloidogenic step of the oligomerization process is the transition from *α*-helix rich (starting from the conformation of APP before being cleavaged) to *β*-sheet rich structures. All atom simulation of A*β*_37−42_ reveals the polymorphism of A*β* oligomers [101]. Replica exchange molecular dynamics simulations were conducted to study the short peptide A*β*_10−35_ [102] and A*β*_16−22_ [103] dimer and trimer formations as well as the A*β*_16−35_ monomer and dimer structure and thermodynamics properties [104]. A coarse-grained model of A*β*_1−42_ was used to study the structural diversity of the dimer [105] in aqueous environment. A single A*β*_40_ peptide was used to study its structural diversity [106] and the relevant effects of insertion depth and ionic strength in the DPPC membrane environment [107]. Further study shows that the thermodynamics and dynamics of A*β* oligomerization are sequence dependent [108]. 

A*β* has two alloforms: one is A*β*_1−40_, the other is A*β*_1−42_. Both have distinct effects and pathways during oligomerization [109,110]. It is generally believed that A*β*_1−40_ peptides are non-amyloidogentic while A*β*_1−42_ are amyloidogentic. Recent studies have shown that A*β* peptides produced in the area with elevated level of cholesterol pose a great risk of Alzheimer’s disease [111] and those genes associated with the cholesterol regulation play a significant role in the predisposition of AD. Here, it raises the question on the effect of cholesterol binding to A*β* peptide and the associated mechanisms of A*β* aggregation in the membrane environment. Recent studies done by our group show that cholesterol molecules compete with the intra-action of A*β* oligomers by binding directly with A*β* peptides. This implies that monomeric A*β* and/or small A*β* aggregations prefer to locate within cholesterol-rich membranes [111,112,113]. Furthermore, A*β* structure evolution in the presence of small and macro-molecules, such as curcumin [114,115], heme [116], resveratrol [115,117], mitoxantrone and pixantrone [118], derivatives of Congo Red [119], 1,4-naphthoquinon-2-yl-L-tryptophan inhibitor [120], EGCG [115,121], NqTrp [115], and inflammation protein complex [122], are also studied. In these studies, A*β* aggregation behaviour is found to be either inhibited or promoted. 

The single mutation of A*β* are also performed to study the mutation effect on A*β* oligomerization process. A2V mutation in A*β*_1−28_ shows that the intrinsic disorder are reduced with a completely different free energy landscape [123]. D7N mutation on the A*β*_40_ and A*β*_42_ exhibits a notable change in secondary structure, final topology and salt bridge compared with wild type [124]. D23N mutation also causes a distinct dimerization pathways compared with wild type in A*β*_1−42_ and A*β*_1−40_ [110]. 

The detailed fibril nucleation and oligomerization are further probed from kinetics and thermodynamics aspect via computational method [125]. 

## 5. Amyloid Fibril Formation and Polymorphism

Since the end of the 20th century, much effort has been made to understand the structure of amyloid fibrils and the mechanism of its formation. With the development of measurement techniques, such as NMR and X-ray crystallography, the polymorphic structures of amyloid fibril have been revealed. The main contribution to the distinct structures of the fibril is the sensitivity of the fibril growth towards the surrounding conditions [126,127,128]. Despite the multiple differences in their overall structures, there exists a common a well-characterized antiparallel *β*-sheets within the fibrils [127]. Molecular dynamics simulations have been employed to investigate the detailed mechanisms of the A*β* fibril formation by adding monomers into the structured oligomers [129]. It was found that the incorporation of the monomers into the oligomers occurs in two distinct stages: the first stage is a rapid conformational change of the monomers from a disordered structure to one with a significant amount of beta-strand content. The second stage is a relatively slow process, namely the docking of the monomer which has adjusted itself into a well-registered antiparallel structure. 

## 6. A*β* Isoforms — Variants of A*β*

The amyloid-cascade hypothesis holds a stronghold in the research of AD. It posits that the process of A*β* aggregation into oligomers and final deposition as plaques is the central pathological events in AD. As stated earlier, A*β*_40_ and A*β*_42_ are two well-recognized isoforms of A*β* being produced. Meanwhile, with the conduct of intensive research in this area, several *C*-terminal truncated isoforms, such as A*β*_43_ [130,131], A*β*_1−15/16_ [132], and carboxyterminally truncated A*β* peptides 1 − 37/38/39 [133], have been revealed and they are suggested to play a crucial role in the AD pathogenesis. In particular, experimental data obtained from both sporadic and familial AD shows that A*β*_43_ is more prevalent than A*β*_40_ in plaque core [130]. 

Interestingly, in AD brains there are a significant proportion of *N*-terminal truncated A*β* variants, such as A*β*_2−17_,A*β*_3−17_ [134], A*β*_*n*−40/42_ with *n* ranges from 2 to 11 [135], pyroglutamate-modified amyloid beta (A*β*_3(*pE*)−42_) and A*β*_11(*pE*)−42_ peptides [136]. Among them the pyroglutamate-modified amyloid beta appears to be the predominant components [137,138,139]. The earliest report of the pyroglutamate-modified amyloid beta-peptides dates back to 1997 and earlier [140,141]. After a decade of inactivity following 1997 and only within the recent five years have some researchers begun to understand their formation [142], structure [143], oligomerization [144], intracellular accumulation [145], and its potential as therapeutic target [138]. At the same time, the first reported peptide in A*β* plaque, the A*β*_4−42_ peptide, has received no attention even though it is as toxic as pyroglutamate-modified amyloid beta peptides and A*β*_42_ [135]. 

## 7. A*β* Oligomers and Tau Protein: Relationship and Link

There are various means by which A*β* oligomers are distributed among cells. These are: diffusion or spread within extracellular parenchymas as oligomers or deposited plaques; adsorption on membrane surface or incorporation into membrane structure forming pores or channels; and accumulation within the neuronal structure. On the other hand, the tau protein is mainly distributed within the intracellular neuron. One possible direct link between the intraneuronal A*β* and tau protein involves the modulation effect between A*β* and tau pathologies [146]. 

Studies have shown that intracellular accumulation appears earlier than amyloid plaque and NFT, and have suggested that intraneuronal A*β* accumulation initiates the caspase-cleavage of tau and precedes the A*β* plaque and NFT formation [147,148]. Meanwhile, tau hyperphosphorylation signal transduction pathways may also be linked indirectly to A*β* oligomers. Recent reviews on the relationship between A*β* pathway and tau pathology can be accessed from references [146,149]. 

## 8. AD Progression Pathway and Current Therapeutic Strategies

Mild cognitive impairment (MCI) has been used to prescribe the transitional stage between healthy brain and dementia. One impairment subtype is amnesic mild cognitive impairment (aMCI), which may increase the risk of progression to AD. Due to a variation in definitions of MCI based on different clinical criteria, the pathology of aMCI still lacks a strong characteristic profile. In terms of the intermediate stages towards AD, MCI shares a lot of similarity with AD, *i.e.*, an increase of NFT in the medial temporal lobe (namely, hippocampus) amygdala. 

In the last few years, donepezil, rivastigmine, and galantamine are prescribed drugs for AD patients to target acetylcholinesterase that inhibits the breaking down of acetylcholine. Another drug memantine has been used to block glutamate receptors against excitotoxicity as a means to cure AD. To date, the acetylcholinesterase inhibitors are the most widely used AD drug and have been to some extent successful in slowing down the process of cognitive impairment [150]. 

## 9. Conclusions

Alzheimer’s disease is a complex and progressive neuro-degenerative disease. There are numerous studies from different points of view on the pathology of AD, such as those mentioned in this review, which involve genetic and environmental factors, tau protein and neurofibrillary tangles, the variety of its isoforms as well as amyloid beta peptides and oligomers. However, all of these issues are not isolated. In all likelihood, the actions among extracellular amyloid *β* peptides and intracellular tau proteins are closely related to each other through a series of complicated, but essentially important, processes and events. Despite the strong links between A*β* and tau protein that have been reported so far, a panorama study of these deeply connected roadmap is still missing. In order to explore the whole landscape of AD, a step by step strategy is of paramount importance, such as the uncovering of the mechanism of A*β* peptide aggregation, which will help to decipher the whole story on the pathogenesis of AD. 
